# Seroprevalence of *Neospora caninum* Infection in Sheep and Goats in Shanxi Province, North China

**DOI:** 10.3390/vetsci13050422

**Published:** 2026-04-26

**Authors:** Dong-Yang Wang, Xun-Zhi Liu, Ze-Dong Zhang, Wen Li, Nan Su, Xing-Quan Zhu, Wen-Wei Gao

**Affiliations:** Shanxi Key Laboratory of Animal Disease Research, Prevention and Control, College of Veterinary Medicine, Shanxi Agricultural University, Jinzhong 030801, China; 17303450339@163.com (D.-Y.W.); liuxunzhi0206@126.com (X.-Z.L.); zhangzedong0519@126.com (Z.-D.Z.); 13934936109@163.com (W.L.); sunan1228@163.com (N.S.); xingquanzhu1@hotmail.com (X.-Q.Z.)

**Keywords:** *Neospora caninum*, seroprevalence, sheep, goats, iELISA, Shanxi Province

## Abstract

*Neospora caninum* is an apicomplexan parasite with a broad geographical distribution and a complex life cycle. It can cause infectious abortions in a variety of animals in the major livestock-producing nations, resulting in huge economic losses to the livestock industry. Shanxi Province in north China is one of China’s important livestock-producing provinces, but the data on the prevalence of *N. caninum* in sheep and goats in this province was not available prior to the present investigation. To fulfill this gap in our knowledge, serum samples were collected from 504 sheep and 300 goats across 11 cities representing three distinct geographical regions of Shanxi Province. A commercially available indirect enzyme-linked immunosorbent assay (iELISA) kit was used to determine the *N. caninum* prevalence by detecting *N. caninum*-specific IgG antibodies. Results showed that a total of 24 sheep (4.8%; 95% CI: 2.9–6.6) and 8 goat samples (2.7%; 95% CI: 0.8–4.5) tested positive for *N. caninum* antibodies. Geographical distribution was identified as the predominant risk factor influencing *N. caninum* infection in sheep and goats in Shanxi Province, with seroprevalence of *N. caninum* ranging from 0% to 16.7% across different sampling sites. This study reports the seroprevalence of *N. caninum* infection in sheep and goats in Shanxi Province for the first time, providing baseline data for the prevention and control of *N. caninum* infection in this northern province of China.

## Simple Summary

*Neospora caninum* is an apicomplexan parasite with a worldwide geographical distribution and a complex life cycle, which causes infectious abortion in various animals and brings huge economic losses to the livestock industry. Shanxi Province is an important livestock-producing province in China, but the prevalence of *N. caninum* in sheep and goats in this province is still unknown. In this study, serum samples were collected from 504 sheep and 300 goats from 11 cities covering three geographical regions of Shanxi Province, and the infection of *N. caninum* was detected by commercial indirect enzyme-linked immunosorbent assay (iELISA). The results showed that the seropositive rates of *N. caninum* in sheep and goats were 4.8% (24/504) and 2.7% (8/300), respectively, and geographical distribution was the main risk factor for infection. This study is the first to report the seroprevalence of *N. caninum* in sheep and goats in Shanxi Province, providing baseline data for the prevention and control of *N. caninum* infection in the province.

## 1. Introduction

*Neospora caninum* is an apicomplexan protozoan with a broad geographical distribution and a complex life cycle, and it is an important parasite that affects many wild and domesticated animals [1]. Despite its close morphological and biological resemblance to *Toxoplasma gondii*, *N. caninum* is recognized as a distinct species with unique host specificity, pathogenic mechanisms, and epidemiological characteristics [2]. The life cycle of *N. caninum* involves both definitive and intermediate hosts, and involves three morphologically distinct stages: tachyzoite, bradyzoite (within tissue cyst) and sporozoite (within oocyst). During the acute phase of infection, rapidly replicating tachyzoites disseminate across multiple organs, while slowly dividing bradyzoites primarily persist in the central nervous system, where they may remain latent until reactivation [3]. Domestic and wild canids have been determined as definitive hosts of *N. caninum*, in which sexual reproduction occurs in the intestinal epithelium, leading to the excretion of environmentally resistant oocysts in feces [2]. Intermediate hosts encompass a broad range of warm-blooded animals, including cattle, sheep, goats and most mammals, in which the parasite undergoes asexual reproduction [2].

Transmission of *N. caninum* mainly occurs through the fecal–oral route via the ingestion of food and water contaminated by sporulated oocysts shed by canids. Vertical transmission is another important route for maintaining the infection cycle, particularly in herds and flocks [4,5]. Up to now, *N. caninum* has been documented worldwide and is considered one of the most important causes of reproductive failure in cattle [1,4]. However, accumulating evidence indicates that *N. caninum* infection in sheep and goats can also result in abortion, placental necrosis, fetal death, stillbirth and other reproductive disorders, leading to significant economic losses [6,7]. As an important parasitic disease, the global economic impact of neosporosis is substantial, with annual losses estimated at over one billion US dollars in the livestock industry [8].

Neosporosis has been recognized as a disease of global veterinary significance. Pathogenically, the overlapping of canids and intermediate hosts is considered as the primary risk factor [3]. Sheep and goats become infected with *N. caninum* by ingesting water or food, or through contact with the environment contaminated with *N. caninum* oocysts which are excreted in canine feces. The seroprevalence of *N. caninum* in sheep and goats varies geographically. For example, *N. caninum* prevalence was 15.5% in sheep and 5% in goats in Egypt [9], 17.2% in sheep and 14% goats in Argentina [10,11], and 17.6% in sheep in Brazil [12], reflecting differences in farm management practices, climatic conditions, the density of definitive hosts, and the diagnostic methods employed. Within China, seroprevalence of *N. caninum* also varies considerably across regions, but data remain scattered and incomplete. The highest seroprevalence in sheep was observed in Qinghai Province (10.33%) [13], followed by Henan Province (7.32%) [14] and Yunnan Province in black-bone sheep (5.69%) [15]. In goats, the highest seroprevalence was reported among black-bone goats (13.61%) and black goats (12.88%) in Yunnan Province [15,16], followed by Qinghai Province (7.23%) [13], Hubei Province (3.9%) [17], Taiwan Province (2.54%) [18], and Hunan Province (2.0%) [13]. These reports showed the epidemiological landscape of *N. caninum* infection in sheep and goats across many provinces of China, whereas that in the northern provinces remains unknown.

Several diagnostic methods have been developed for the detection of *N. caninum* infection, including histopathological examination, immunohistochemistry, polymerase chain reaction (PCR) and serological assays [1,5]. Among serological methods, the indirect fluorescent antibody test (IFAT) is commonly used as a reference standard, while enzyme-linked immunosorbent assays (ELISA), including indirect ELISA (iELISA) and competitive ELISA (cELISA), have gained widespread applications in epidemiological surveys due to their high throughput and suitability for large-scale screening [5,19]. So far, there are no effective drugs or vaccines to block the spread of neosporosis that causes abortion, although some compounds have been shown to be effective against *N. caninum* tachyzoite in vitro, but there is still a challenge to cure encysted bradyzoites [6].

Shanxi Province, located in northern China, is an important livestock-producing region with a substantial population of sheep and goats. However, there is no data on the prevalence of *N. caninum* in sheep and goats in Shanxi Province. Given the economic importance of small ruminant husbandry in Shanxi Province and the absence of specific drugs or vaccines against *N. caninum*, it is important to obtain baseline epidemiological data of *N. caninum* in sheep and goats in Shanxi Province. Therefore, an indirect inhibition enzyme-linked immunosorbent assay (iELISA) was employed in the present study to detect *N. caninum*-specific IgG antibodies in serum samples collected from sheep and goats across 11 cities in Shanxi Province, and associated risk factors were analyzed, providing a scientific basis for targeted prevention and control strategies.

## 2. Materials and Methods

### 2.1. Sampling Sites and Serum Samples Collection

Based on the reported prevalence of *N. caninum* in goats and sheep in China, the minimum representative sample size required for this study was estimated to be 31 using the Thrusfield’s [20] formula *n* = [1.96^2^ × *P*_exp_(1 − *P*_exp_)]/d^2^ (*n* = the required sample size; *P*_exp_ = expected prevalence; d = desired absolute precision). After informed consent had been obtained from the farm owners, serum samples were collected from the sheep or goats in the farms using a systematic random sampling method.

**Figure 1:**
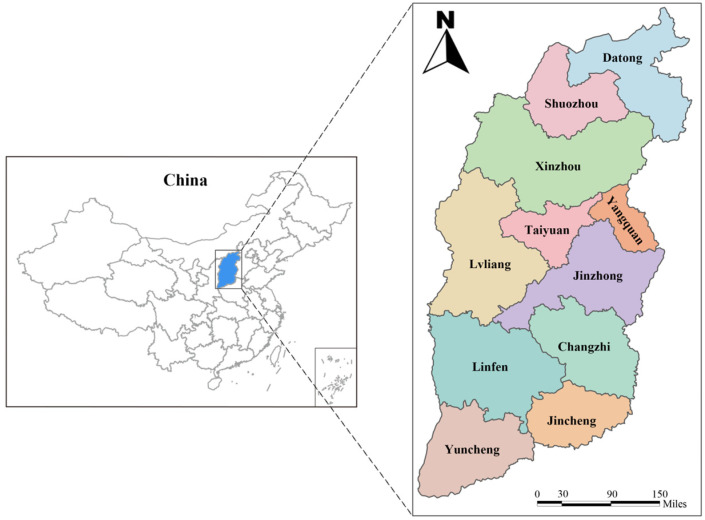
Locations for sampling serum samples of sheep and goats in Shanxi Province, China.

In March 2024, a total of 504 sheep serum samples and 300 goat serum samples were collected randomly from 40 farms in 11 cities located in the northern, central and southern regions of Shanxi Province (Figure 1). Approximately 5 mL of blood was collected aseptically from the jugular vein of each animal using sterile vacuum blood collection tubes without anticoagulant. The samples were allowed to clot at room temperature for 4 h and were centrifuged at 4000× *g* for 10 min to separate the serum, and then the separated serum was transported to the laboratory where it was stored at −20 °C until serological analysis. All pertinent animal metadata, including species and sampling site, were recorded at the time of sampling.

### 2.2. Serological Examination

Following the manufacturer’s instructions, serum samples were tested for *N. caninum*-specific IgG antibodies using the ID Screen^®^ *Neospora caninum* Indirect ELISA kit (Innovative Diagnostics, Grabels, Hérault, France). To ensure the accuracy of the experiment, all reagents and samples were equilibrated to room temperature (approximately 20 °C) and mixed gently prior to use. Subsequently, optical density (OD) was measured at 450 nm with a CMax Plus microplate reader (Molecular Devices, Sunnyvale, CA, USA). Standard positive and negative controls from the kit were set in each ELISA detection. For the assay to be considered valid, the mean OD of the positive control (OD_PC_; detected) was required to exceed 0.350, and the OD_PC_/OD_NC_ ratio (where OD_NC_ represents the negative control OD) had to be greater than three. The sample-to-positive percentage (S/P%) was calculated as [(Sample OD − OD_NC_)/OD_PC_ − OD_NC_] × 100%. Samples were interpreted as positive if S/P% ≥ 50%, negative if S/P% ≤ 40%, and suspect if 40% < S/P% < 50%. Suspect samples were retested once; if the retest yielded a definitive result (positive or negative), that result was adopted.

### 2.3. Statistical Analysis

The data obtained in this study were analyzed for the relevance between variables (e.g., geographical region) and *N. caninum* seroprevalence using the Chi-square (χ^2^) test in SPSS 26.0 (SPSS, Inc., Chicago, IL, USA). Odds ratios (OR) and 95% confidence intervals (95% CI) were calculated to assess the strength of associations. The variable with *p* < 0.05 was considered statistically significant.

## 3. Results

### 3.1. Seroprevalence of N. caninum in Sheep and Goat in Shanxi Province

In this study, a total of 504 sheep serum samples and 300 goat serum samples from 11 cities were tested for *N. caninum* antibodies. Overall, 24 sheep and 8 goat samples were determined as seropositive, with a total *N. caninum* seroprevalence of 4.8% (95% CI: 2.9–6.6) in sheep and 2.7% (95% CI: 0.8–4.5) in goats, respectively. Among the 11 sampling cities, significantly varied seroprevalence was observed ranging from 0% to 16.7% (Table 1). In northern Shanxi Province, the highest seroprevalence was detected in Datong city, with 16.7% of *N. caninum* in sheep and 15.0% in goats, respectively. In Shuozhou city, 2.5% of sheep were positive for *N. caninum* (95% CI: 0–7.3), while no goats tested seropositive. In Xinzhou city, *N. caninum* seroprevalence in goats was 3.8% (95% CI: 0–7.9), and no positive sample was detected in sheep. In central Shanxi Province, no positive samples were detected in Taiyuan city or Yangquan city. In Jinzhong city, 7.5% of sheep were *N. caninum*-positive (95% CI: 0–15.7), with no *N. caninum*-positive samples detected in goats. In Lvliang city, no sheep were detected as positive for *N. caninum*, but *N. caninum* seroprevalence in goats was 5% (95% CI: 0–11.8). In southern Shanxi Province, no positive serum sample was detected in sheep or goats from Changzhi city and Linfen city. Totally, 7.5% of sheep in Jincheng city (95% CI: 1.7–13.3) and 6.7% of sheep in Yuncheng city (95% CI: 0.4–13.0) were detected as *N. caninum*-positive, but no goat samples tested as *N. caninum*-positive in either city (Table 1).

Regionally, the highest *N. caninum* seropositivity was detected in northern Shanxi Province, with 11 positive samples in sheep (10 from Datong city and one from Shuozhou city) and six positive samples in goats (three each from Datong city and Xinzhou city), corresponding to a regional *N. caninum* seroprevalence of 8.5% (95% CI: 4.6–12.4). In central Shanxi Province, three positive samples in sheep (all from Jinzhong city) and two positive samples in goats (from Lvliang city) were detected, with an overall *N. caninum* prevalence of 1.6% (95% CI: 0.2–3.1). In southern Shanxi Province, 10 positive samples in sheep (6 from Jincheng city and 4 from Yuncheng city) and zero positive samples in goats were recorded, with a total prevalence of 3.3% (95% CI: 1.3–5.4) (Table 2).

### 3.2. Analysis of Risk Factors

Table 2 shows the results of the statistical analysis of risk factors for *N. caninum* seroprevalence between geographical distribution and species. Geographical location was identified as a key risk factor (*p* < 0.001). In northern Shanxi, 17 of 200 samples were positive for *N. caninum* (8.5%, 95% CI: 4.6–12.4), and the seroprevalence of *N. caninum* was significantly higher than that in central Shanxi (OR = 5.6, 95% CI: 2.0–15.3). In southern Shanxi Province, 10 out of 300 samples were detected as *N. caninum*-positive (3.3%; 95% CI: 1.3–5.4), which was not significantly different from that in central Shanxi Province (*p* = 0.26). However, the risk of infection in the southern region was higher compared to the northern region (OR = 3.1, 95% CI: 0.6–22.7).

However, the difference in *N. caninum* seroprevalence between sheep and goats was not statistically significant (*p* = 0.048), indicating that animal species was not a major determinant of *N. caninum* seroprevalence in this study. Nevertheless, a higher *N. caninum* seroprevalence was detected in sheep than in goats (OR = 1.9, 95% CI= 0.9–4.3). For sheep, *N. caninum* seroprevalence differed significantly across study areas (*p* < 0.001). In northern Shanxi, 11.0% of serum samples in sheep were positive for *N. caninum*, indicating a substantially higher infection risk than that in the Central Shanxi Province (OR = 7.5, 95% CI: 2.0–27.4). Otherwise, the risk of *N. caninum* infection in sheep in southern Shanxi Province was 1.69-fold higher than in central Shanxi Province (95% CI: 0.4–6.9).

## 4. Discussion

*N. caninum* is a globally distributed protozoan parasite that causes substantial economic losses to the livestock industry. As intermediate hosts of *N. caninum*, ruminants are infected with *N. caninum* primarily through ingestion of food or water contaminated with oocysts shed by canids, or through vertical transmission from infected dams. A recent meta-analysis revealed that *N. caninum* seropositive doe goats were 3.1 times more likely to abort compared with seronegative animals (OR = 3.1, 95% CI: 1.0–9.2) [21]. Studies have demonstrated that *N. caninum* infection can be vertically transmitted in sheep and is associated with fetal loss, abortion, and neonatal mortality [4,7,22]. Current knowledge regarding *N. caninum* seroprevalence in sheep and goats in China remains limited, and a better understanding of *N. caninum* prevalence is essential for developing effective prevention and control strategies.

In this study, the seroprevalence of *N. caninum* was determined to be 4.8% in sheep and 2.7% in goats using an indirect ELISA (iELISA). Compared with other provinces of China, *N. caninum* seroprevalence in sheep in Shanxi Province (4.8%) was lower than that in Qinghai Province (10.33%) [13], Gansu Province (8.4%) [23], Henan Province (7.32%), [14] and black-bone sheep in Yunnan Province (5.69%) [15]. In goats, *N. caninum* seroprevalence in Shanxi Province (2.7%) was lower than that in Yunnan Province among black-bone goats (13.61%) [15], and black goats (12.88%) [14], Henan Province (7.32% and 2.8%) [14,24], and Hubei Province (3.9%) [17], but comparable to that in Taiwan Province (2.54%) [18] and Hunan Province (2.0%) [25]. These differences may be related to variations in ecological conditions, farming management systems, the density and proximity of canine populations, and the specific diagnostic assays employed across different studies [13,20]. Notably, in Qinghai Province, the presence of dogs, the pasturing system, herd size, and farm hygiene were identified as significant risk factors for *N. caninum* seroprevalence [13], whereas the present study was limited to geographical location and species as variables. The relatively lower *N. caninum* seroprevalence in Shanxi Province may also partly be attributed to the predominant intensive or semi-intensive farming systems in this region, which could reduce horizontal transmission opportunities compared with the extensive pastoral systems more commonly practiced in western China [13,14].

Globally, *N. caninum* seroprevalence in small ruminants varies considerably across countries, regions, and individual flocks. A previous systematic review indicated that including 35,740 sheep from 30 countries estimated a global pooled seroprevalence of 13.0% (95% CI: 10–15) [10]. Likewise, another meta-analysis showed a 6.0% (95% CI: 4.4–7.8) seroprevalence of *N. caninum* in goats worldwide [20]. The present study found *N. caninum* seroprevalences of 4.8% and 2.7% in sheep and goats, respectively, which were lower than those in most countries, such as sheep in Mexico (21.0%) [26], sheep (17.2%) and goats (14%) in Argentina [11], goats in Ecuador (12.11%) [27], sheep (0.9–9.9%) and goats (6.2%) in Iran [27], and goats and sheep in Somalia (both 2.2%) [28]. These considerable variations across countries may be related to differences in geographical and climatic conditions, livestock management systems, definitive host population density, diagnostic methodologies, and sample sizes.

Furthermore, molecular studies have provided additional insights into the role of *N. caninum* in reproductive failure. A recent systematic review and meta-analysis reported that the pooled prevalence of *N. caninum* in aborted fetuses is 15% (95% CI: 9–21%) for sheep and 7% (95% CI: 2–12%) for goats, while the seroprevalence in ovine aborted fetuses and sheep that experienced abortion was estimated at 17% and approximately 3%, respectively [1]. The total *N. caninum* seroprevalence in small ruminants detected in this study was 4.0% (95%CI: 2.6–5.3). The mild *N. caninum* prevalence reflects the absence of a large-scale outbreak of neosporosis, indicating favorable conditions for early intervention.

In addition, the present study explored the associated risk factors among *N. caninum* seroprevalence in study areas. Univariate analysis identified geographical location as a significant risk factor (*p* < 0.001), with a spatial distribution difference. Northern Shanxi showed the highest *N. caninum* seroprevalence (8.5%; 95% CI: 4.6–12.4), followed by southern Shanxi (3.3%, 95% CI: 1.3–5.4) and central Shanxi (1.6%; 95% CI: 0.2–3.1), with 5.6-fold and 2.1-fold increases in chances of *N. caninum* infection in northern and southern areas than in the central area, respectively (Table 2). This finding is consistent with previous studies that have identified geographical location as a significant determinant of *N. caninum* seroprevalence in small ruminants [13,15,20,29]. Transmission of *N. caninum* relies on canine definitive hosts that shed oocysts into the environment [30]. Datong city, a northern transportation hub with higher human and domestic/stray dog densities in peri-urban areas, likely presents greater environmental contamination with oocysts due to dog activity, increasing pasture and water source exposure risk. Previous studies have demonstrated that the presence of dogs with flocks significantly increased seropositivity [12,20,31]. In this study, no data of dog density around the sampled farms was recorded to assess the association between dogs and *N. caninum* infection. However, all sampled farms operated under a semi-intensive management system, which may increase the opportunities for transmission of *N. caninum* by dogs. In southern Shanxi Province, Jincheng (7.5%) and Yuncheng (6.7%) emerged as higher-prevalence areas, whereas Taiyuan city, Yangquan city, Changzhi city, and Linfen city showed no *N. caninum*-seropositive samples in this study. This marked intra-regional variation further underscores the decisive role of geographical location in shaping infection distribution.

No significant difference in *N. caninum* seroprevalence was observed between sheep and goats in the present study [*p* = 0.142], further highlighting the greater importance of geographical factors than species factor in its transmission. Consistent with most literature, sheep showed a higher susceptibility to *N. caninum* infection than goats [11,12,15,21,32], though the biological basis for this difference remains to be elucidated. This species-dependent difference has been attributed to the distinct feeding behaviors of sheep and goats: sheep are typically grazers that forage close to the ground and are therefore more likely to ingest oocysts from contaminated pasture, whereas goats are predominantly browsers that feed on elevated vegetation, thereby reducing their exposure to ground-level contaminants [33,34]. However, in some studies, no significant difference has been found between the two species, and goats have occasionally shown higher seroprevalence [26,28], indicating that local environmental and management factors may override inherent species differences in susceptibility.

Two limitations of this study should be acknowledged. First, additional potential risk factors, such as age, farm management practices, the presence of dogs on farms and reproductive history were not explored due to the lack of available metadata. Second, the relatively small sample sizes may have limited the statistical strength between prevalence and risk factors. Future studies with larger sample sizes, broader geographical coverage and more risk factor assessments are warranted to further characterize the epidemiology of *N. caninum* in small ruminants in Shanxi Province.

## 5. Conclusions

This study detected a *N. caninum* seroprevalence of 4.8% (24/504) in sheep and 2.7% (8/300) in goats in the Shanxi Province, north China. Geographical location was identified as the primary risk factor associated with *N. caninum* infection. The findings of the present cross-sectional investigation provided baseline data for establishing effective control and prevention strategies to minimize the economic losses associated with *N. caninum* infections in sheep and goats in Shanxi Province. Further studies should expand the sampling regions, increase sample size and evaluate additional risk factors to achieve a more comprehensive understanding of *N. caninum* epidemiology in Shanxi Province, north China.

## Figures and Tables

**Table 1:** Seroprevalence of *Neospora caninum* in sheep and goats in Shanxi Province, North China.

Geographical Location	City	Sheep				Goats			
		Sample Size	Positive Samples	Prevalence % (95% CI)	*p*-Value	Sample Size	Positive Samples	Prevalence % (95% CI)	*p*-Value *
North Shanxi	Datong	60	10	16.7 (7.2–26.1)	0.060	20	3	15.0 (0–30.7)	0.048
	Shuozhou	40	1	2.5 (0–7.3)		-	-	–	
	Xinzhou	-	-	–		80	3	3.8 (0–7.9)	
	Subtotal	100	11	11.0 (4.9–17.1)		100	6	6.0 (1.4–10.7)	
Central Shanxi	Taiyuan	64	0	0	0.028	-	-	–	0.241
	Yangquan	40	0	0		40	0	0	
	Jinzhong	40	3	7.5 (0–15.7)		40	0	0	
	Lvliang	40	0	0		40	2	5.0 (0–11.8)	
	Subtotal	184	3	1.6 (0–3.5)		120	2	1.7 (0–4.0)	
South Shanxi	Changzhi	80	0	0	0.035	-	-	–	NA
	Linfen	-	-	–		80	0	0	
	Jincheng	80	6	7.5 (1.7–13.3)		-	-	–	
	Yuncheng	60	4	6.7 (0.4–13.0)		-	-	–	
	Subtotal	220	10	4.6 (1.8–7.3)		80	0	–	
Total		504	24	4.8 (2.9–6.6)		300	8	2.7 (0.8–4.5)	

*: *p*-values were calculated only for variables with valid observations to evaluate statistical significance.

**Table 2:** Geographical distribution of *N. caninum* seroprevalence in sheep and goats in Shanxi Province.

Categories	Areas	Sample Size	Positive	Prevalence (95% CI)	*p*-Value	OR
Geographic location	North Shanxi	200	17	8.5 (4.6–12.4)	<0.001	5.6 (2.0–15.3)
	Central Shanxi	304	5	1.6 (0.2–3.1)		Reference
	South Shanxi	300	10	3.3 (1.3–5.4)		2.1 (0.7–6.1)
Species	Sheep	504	24	4.8 (2.9–6.6)	0.048	1.9 (0.9–4.3)
	Goat	300	8	2.7 (0.8–4.5)		Reference
Sheep	North Shanxi	100	11	11.0 (4.9–17.1)	<0.001	7.5 (2.0–27.4)
	Central Shanxi	184	3	1.6 (0–3.5)		Reference
	South Shanxi	220	6	2.7 (0.6–4.9)		1.7 (0.4–6.9)
Goat	North Shanxi	100	6	6.0 (1.4–10.7)	0.26	3.1 (0.6–22.7)
	Central Shanxi	120	2	1.7 (0–4.0)		Reference
	South Shanxi	80	0	0		-

## Data Availability

The original contributions presented in this study are included in the article. Further inquiries can be directed to the corresponding author.
